# Appropriateness of antibiotic use in nursing homes for suspected urinary tract infections: comparison across five European countries

**DOI:** 10.1007/s41999-025-01185-0

**Published:** 2025-03-27

**Authors:** Matilde Bøgelund Hansen, Jesper Lykkegaard, Malene Plejdrup Hansen, Carl Llor, Ana Garcia Sangenis, Pia Touboul-Lundgren, Pascale Bruno, Ruta Radzeviciene, Lina Jaruseviciene, Marilena Anastasaki, Christos Lionis, Anna Kowalczyk, Maciek Godycki-Ćwirko, Beatriz Gonzalez Lopez-Valcárcel, Fabiana Raynal Floriano, Athina Chalkidou, Lars Bjerrum, Jette Nygaard Jensen

**Affiliations:** 1 Department of Clinical Microbiology, Copenhagen University Hospital–Herlev and Gentofte, Copenhagen, Denmark; 2 Audit Project Odense, Research Unit of General Practice, University of Southern Denmark, Odense, Denmark; 3 Center for General Practice, Aalborg University, Aalborg, Denmark; 4 Institut Català de La Salut, Via Roma Health Centre, C. Manso, 19, 3rd Floor, 08015 Barcelona, Spain; 5 Fundació Institut Universitari Per a La Recerca a L’Atenció Primària de Salut Jordi Gol, Barcelona, Spain; 6 Department of Public Health, Nice University Hospital, Nice, France; 7 Ltd. Mano Seimos Gydytojas (My Family Doctor), Klapeida, Lithuania; 8 Lithuanian University of Health Sciences, Kaunas, Lithuania; 9 Clinic of Social and Family Medicine, School of Medicine, University of Crete, Rethymn, Greece; 10 Centre for Family and Community Medicine, The Faculty of Health Sciences, The Medical University of Lodz, Lodz, Poland; 11 University of Las Palmas de Gran Canaria, Las Palmas, Spain; 12 Section of General Practice and Research Unit for General Practice, Department of Public Health, University of Copenhagen, Copenhagen, Denmark; 13 Capital Region Committee for Antimicrobial Stewardship and Prevention of Hospital Infections, Hillerød, Denmark

**Keywords:** Antimicrobial stewardship, Medical audit, Quality of health care, Clinical decision-making, Urinary tract infections, Nursing homes

## Abstract

**Purpose:**

Antibiotic use is the main driver of bacterial antimicrobial resistance. Urinary tract infections (UTIs), for which antibiotics are often prescribed, are among the most common infections among residents in nursing homes. This study aimed to estimate and compare the appropriateness of antibiotic use for suspected UTIs among nursing home residents across five European countries. Both the decision to treat and the choice of antibiotics used were explored.

**Methods:**

This cross-sectional study used the Audit Project Odense (APO) method. The appropriateness of the treatment decision for UTIs was evaluated according to symptom presentation in residents without indwelling urinary catheters. The choice of antibiotic used for treatment was evaluated according to the first-line antibiotic recommended for the treatment of UTIs in the respective countries. Data were collected through an institutional sign-up questionnaire and a clinical case registration chart. All residents in new or ongoing treatment with systemic antibiotics were registered during a 6-week period between February and April 2022.

**Results:**

A total of 70 nursing homes or wards registered 508 antibiotic treatments for suspected UTIs. The proportion of antibiotics prescribed for UTIs to nursing home residents with no specific UTI symptoms varied from 10% in Poland to 68% in Spain. The proportion of treatments with the first-line choice of antibiotics for UTIs also varied from 28% in France and Poland to 55% in Spain.

**Conclusion:**

There was a substantial proportion of potentially inappropriate antibiotic treatments, with notable variation among the countries studied.

**Supplementary Information:**

The online version contains supplementary material available at 10.1007/s41999-025-01185-0.

## Key summary points

**Aim:**

To estimate and compare the appropriateness of antibiotic use for suspected UTIs (Urinary tract infections) among nursing home residents across five European countries.

**Findings:**

The decision to start antibiotics for UTIs in residents without catheters was deemed to be highly inappropriate for almost half of the treatments. A first-line antibiotic was administered for UTIs in nursing home residents for less than half of the antibiotic treatments.

**Message:**

There is significant differences in antibiotic prescribing practices for suspected urinary tract infections in nursing homes across five European countries, and our findings indicate a substantial proportion of inappropriate antibiotic treatment.

**Supplementary Information:**

The online version contains supplementary material available at 10.1007/s41999-025-01185-0.

## Introduction

Antibiotic use is the main driver of bacterial antimicrobial resistance, which is a critical global health concern [1, 2]. The European Commission guidelines for prudent use of antibiotics in humans specify that nursing homes should target identified areas of antimicrobial overuse and misuse [3].

Studies have investigated antibiotic use in nursing homes with a particular focus on the quality of diagnosis and appropriateness of antibiotic selection, and the results indicate that the guidelines for antibiotic treatment are not adequately followed [4–8]. There are several reasons why it is particularly difficult to promote appropriate antibiotic use in nursing homes. Diagnostic resources may be limited, and medical decision-making can be challenging in frail nursing home populations [9, 10].

Urinary tract infections (UTIs) are among the most common infections among nursing home residents, for which antibiotics are often prescribed [11, 12] and the appropriateness of initiating antibiotic treatment for UTIs only meets the treatment criteria among 8–44% of nursing home residents with suspected UTIs [13].

Antibiotic use in nursing homes varies significantly across European countries, with less than 20% of the variation explained by nursing home and resident characteristics [11]. National or regional regulations and guidelines as well as prescriber habits and preferences appear to play a significant role in antibiotic use [11]. Although studies have compared antibiotic use across nursing homes in Europe, to our knowledge, no previous study has compared the appropriateness of antibiotic use for suspected UTIs in nursing homes across European countries. Such information is needed to inform the implementation of an antibiotic stewardship program [14]. Therefore, this study aimed to estimate and compare the appropriateness of antibiotic use for suspected UTIs among nursing home residents across five European countries. The decision to treat and choice of antibiotics used were explored.

## Materials and methods

### Design

This cross-sectional study used the Audit Project Odense (APO) method, which is a prospective self-registry methodology [15]. The APO methodology was developed for quality improvement and encompasses an audit registration for healthcare professionals to register key variables related to diagnosis and treatment in a prespecified chart. This study is part of the Health Alliance for Prudent Prescription and Yield of Antibiotics in a Patient-Centered Perspective (HAPPY PATIENT) project and aims to implement EU guidelines for the prudent use of antimicrobials in humans across four healthcare settings (general practice, out-of-hour services, pharmacies, and nursing homes). Detailed information regarding the study method can be found in the study protocol [16].

### Setting

This study included nursing homes in five European countries: France, Greece, Poland, Spain, and Lithuania. The countries were selected because there are variations in antibiotic use [17] and different organizations of health care that ensure better generalization of the results [16].

For this study, the nursing homes were recruited by a national coordinator in each of the five countries via a snowball recruitment strategy. The national coordinator contacted nursing homes by reaching professional organizations for older adults and by personal contacts/visits to the nursing home. Nursing homes or individual wards with nursing home residents within institutions were eligible to participate. Thus, the participating institutes were either entire nursing homes or wards within larger institutions. Each participating unit was independent in the sense that one unit (nursing home or ward) was defined as a group of healthcare professionals that worked as a team around the same residents.

### Data collection

Data were collected via a sign-up questionnaire and a registration chart. Each participating nursing home completed the sign-up questionnaire, which included a consent form, as well as a background questionnaire about nursing home characteristics (Supplementary file 1). The development of the registration chart has been described in the study protocol [16]. The registration chart allowed nursing homes to anonymously register all residents in new or ongoing treatment with systemic antibiotics during a 6-week period between February and April 2022. The registration chart (Supplementary file 2) was distributed to the nursing homes, along with an instruction document. Each antibiotic treatment given to a nursing home resident was registered on the chart, along with information on age, sex, focus of infection (urinary/respiratory/skin/other), symptoms (nonspecific and urogenital symptoms), indwelling urinary catheter status, urine tests, antibiotic treatment, type of treatment (startup/prevention), setting of treatment initiation (nursing home/hospital), treatment duration, and perceived demand for antibiotics. Age and duration of symptoms were counted in whole numbers, and all variables were ticked off if considered present by the healthcare professional; otherwise, the option ‘none of the above’ was filled in as informed in the instructions. On the first day of the registration period, the healthcare professional registered all current antibiotic treatments and, consequently, registered antibiotic treatments if they were initiated. A country-specific list of antibiotics was provided to support the staff in recording the correct antibiotic class for the brand names of common types of antibiotics in the chart. The charts were returned by postal courier and/or digital scans to the national coordinators, who transferred them to European coordinators.

### Data analyses

All antibiotic treatments for suspected UTIs were included in the analysis. The appropriateness of the antibiotic treatment decision for UTIs was evaluated according to symptom presentation in residents without an indwelling urinary catheter, in line with the findings of a Delphi study on the treatment of suspected UTIs in frail older adults [18]. Thus, we considered the symptoms of dysuria, urgency, frequency, and incontinence as symptoms of lower UTI and flank/back pain as symptoms of upper UTI. The consortium of the HAPPY PATIENT project determined that the appropriateness of antibiotic treatment should be evaluated using four categories: highly appropriate, appropriate, likely inappropriate, and highly inappropriate. These categories incorporate signs and symptoms reflecting the findings of the study by van Buul [18]: Antibiotic treatment in residents with two or more lower UTI symptoms or flank/back pain alone was considered highly appropriate. Antibiotic treatment in residents with one lower UTI symptom in combination with fever or shaking chills (systemic symptoms) was considered appropriate, whereas treatment was considered likely inappropriate in residents with only one lower UTI symptom. Antibiotic treatment in residents without lower or upper UTI symptoms is considered highly inappropriate. We focused on the presentation of UTI-specific symptoms since many conditions in frail older patients present atypically, and it is well established that UTI should be diagnosed only when there are new-onset localizing genitourinary signs and symptoms [19].

The following first-line antibiotics were considered for cystitis in each country: Spain and Lithuania: fosfomycin/nitrofurantoin; Greece and Poland: nitrofurantoin/fosfomycin and trimethoprim + sulfamethoxazole; and France: fosfomycin/amoxicillin.

The appropriateness of the choice of antibiotics for suspected UTIs was evaluated according to the first-line antibiotics used for treating cystitis in the respective countries. The first-line antibiotic is the initial recommended antibiotic according to local and national guidelines. Recommendations are based on local antibiotic resistance patterns, which differ across countries.

The data were analyzed via IBM SPSS Statistics for Windows Version 29.0.1.0 [22]. To evaluate the appropriateness of the antibiotic treatment decision, we included all start-up antibiotic treatments for UTIs in residents without an indwelling urinary catheter. To evaluate the choice of antibiotics for the treatment of UTIs, we included all start-up antibiotic treatments in residents with and without an indwelling urinary catheter. We only included start-up antibiotic treatments and omitted preventive treatments as well as antibiotic treatments that were given as a prolongation of the current antibiotic treatment. To adjust for age and to assess differences in the appropriateness of the antibiotic start-up treatment between the countries, a linear mixed model regression analysis was conducted. The outcome variable, representing the appropriateness of the antibiotic treatment start-up treatment decision, was treated as a continuous variable with values from 1 to 4 (1 = highly appropriate, 2 = appropriate, 3 = inappropriate, and 4 = highly inappropriate). A linear mixed model was chosen to account for the clustering of observations within nursing homes by specifying a random intercept model. Statistical significance was set at *p* < 0.05.

## Results

### Description of the participating nursing homes

A total of 79 nursing homes or nursing home wards participated in the study. Nine did not return any filled-in registration charts, and the data were, therefore, based on 70 nursing homes or nursing home wards. The total number of nursing home residents was + 5260 (3 of 19 participating institutes in Lithuania did not provide the total number of residents), with 1204 residents in Spain, 1174 residents in France, 346 residents in Greece, 1371 residents in Poland, and 1165 + residents in Lithuania. The characteristics of the participating nursing homes and wards are summarized in Table 1. The results indicated that nursing home characteristics differed both within and across the five participating countries. Compared with those in France and Spain, nursing homes in Lithuania and Poland had more residents and more often had public ownership and available nursing care. With respect to antibiotic prescribing decisions, in Lithuania, antibiotics are almost always prescribed by a doctor related to a specific nursing home. However, in the other four countries, antibiotics were also prescribed by doctors who were not necessarily related to the nursing homes. Teleconsultations were more common in Spain than in other countries where the consultation was often face-to-face. In Poland, very few participating institutes reported that they performed a urine dipstick test. In the other four countries, urine dipsticks were often or sometimes performed in most participating nursing homes.

**Table 1:** Characteristics of the participating nursing homes, by country, 2022, *n* = 70

	France *N* = 11		Greece *N* = 5		Lithuania *N* = 19		Poland *N* = 17		Spain *N* = 18		Total *N* = 70	
Number of participating institutes												
Entire nursing homes	11		5		4		9		11		40	
Individual wards within the nursing home	0		0		15		8		7		30	
Missing	0		0		0		0		0		0	
Total	11		5		19		17		18		70	
Number of residents (entire nursing home), Median (IQR)	87	(66–111)	72	(71–78)	263	(188–298)	89	(67–160)	79	(70–92)	83	(66–130)
Number of residents (individual wards), Median (IQR)	N/A		N/A		23	(18–30)	54	(52–55)	35	(32–42)	32	(25–51)
Background of health care professional in charge of audit												
Physician	7		1		15		3		11		37	
Nurse	2		3		0		11		7		23	
Other	2		1		1		3		0		7	
Missing	0		0		3		0		0		3	
Total	11		5		19		17		18		70	
Nursing home ownership												
Public	6		2		19		14		10		51	
Private	2		2		0		1		7		12	
Not-for-profit	2		1		0		0		0		3	
None of the above	1		0		0		2		1		4	
Missing	0		0		0		0		0		0	
Total	11		5		19		17		18		70	
Nursing care available 24 h a day												
Yes	4		5		19		16		5		49	
No	7		0		0		1		13		21	
Missing	0		0		0		0		0		0	
Total	11		5		19		17		18		70	
Resident population												
Lower dependency level	2		1		0		2		3		8	
High dependency level	2		1		11		1		4		19	
Mixed dependency level	7		3		6		12		11		39	
Specific needs of care (e.g. psychiatric illnesses, rehabilitation care)	0		0		0		1		0		1	
Other	0		0		2		1		0		3	
Missing	0		0		0		0		0		0	
Total	11		5		19		17		18		70	
Antibiotics prescribed by												
External doctor	6		1		2		8		3		20	
Doctor employed by the nursing home	0		2		17		8		6		33	
External or internal	5		2		0		1		9		17	
Without prescription	0		0		0		0		0		0	
Missing	0		0		0		0		0		0	
Total	11		5		19		17		18		70	
Type of contact with the physician*												
Tele-consultation	0		2		0		5		13		20	
Onsite consultation with a physician	0		0		2		3		3		8	
Onsite consultation at the nursing home	10		5		18		15		18		66	
Other	1		0		0		0		1		2	
Missing	0		0		0		0		0		0	
Use of the urine dipstick test												
Routinely	4		0		14		0		18		32	
Sometimes	7		2		4		1		0		14	
Never	0		2		0		9		0		11	
Missing	0		1		1		7		0		13	
Total	11		5		19		17		18		70	

*Several answers for each participating nursing home possible

### Description of antibiotic use for urinary tract infection and the resident population

A total of 1211 antibiotic treatments were registered among the nursing home residents (Table 2). The infection types for which antibiotics are administered vary across countries; however, UTIs are the most common indication for antibiotic use. Overall, three out of four antibiotic treatments for UTIs were administered to female residents, and 52% of the residents who received antibiotics for UTIs were aged ≥ 85 years. The sex and age distributions of the residents receiving antibiotic treatment for UTIs varied across countries, with ages ranging from 23 to 107 years (mean age 84, median age 83). Compared with other countries, Lithuania and Poland had a greater proportion of males under 85 years of age who received antibiotic treatment for UTIs. One of the four antibiotic treatments for UTIs was administered to residents with an indwelling urinary catheter. Marked differences in the presence of an indwelling urinary catheter among residents receiving antibiotics for UTIs were observed across countries: less than 10% of residents in France and Spain and approximately 40% in Lithuania and Poland.

**Table 2:** Description of antibiotic treatment for UTIs and characteristics of residents receiving antibiotics

	France		Greece		Lithuania		Poland		Spain		Total	
	n	(%)	n	(%)	n	(%)	n	(%)	n	(%)	n	(%)
Total antibiotic use	135		73		176		428		399		1211	
Here antibiotic use for UTI	55	(40.7)	17	(23.3)	43	(24.4)	156	(36.4)	237	(59.4)	508	(41.9)
Age of resident receiving antibiotic for UTI												
Under 85	10	(18.2)	4	(23.5)	34	(79.1)	120	(76.9)	76	(32.1)	244	(48.0)
85 +	45	(81.8)	13	(76.5)	9	(20.9)	36	(23.1)	161	(67.9)	264	(52.0)
Total	55	(100)	17	(100)	43	(100)	156	(100)	237	(100)	508	(100)
Sex												
Female	45	(81.8)	13	(76.5)	26	(60.5)	96	(61.5)	199	(84.0)	379	(74.6)
Type of treatment												
Start-up treatment	47	(85.5)	14	(82.4)	32	(74.4)	134	(85.9)	215	(90.7)	442	(87.0)
Prophylaxis	2	(3.6)	3	(17.6)	1	(2.3)	4	(2.6)	8	(3.4)	18	(3.5)
Extension of current treatment	6	(10.9)	0	(0)	9	(20.9)	16	(10.3)	10	(4.2)	41	(8.1)
Unknown	0	(0)	0	(0)	0	(0)	2	(1.3)	3	(1.3)	5	(1.0)
Missing	0	(0)	0	(0)	1	(2.3)	0	(0)	1	(0.4)	2	(0.4)
Total	55	(100)	17	(100)	43	(100)	156	(100)	237	(100)	508	(100)
Indwelling urinary catheter												
Yes	5	(9.1)	14	(82.4)	17	(39.5)	71	(45.5)	20	(8.4)	127	(25)
No	50	(90.9)	3	(17.6)	23	(53.5)	85	(54.5)	215	(90.7)	376	(74.0)
Missing	0	(0)	0	(0)	3	(7)	0	(0)	2	(0.8)	5	(1)
Total	55	(100)	17	(100)	43	(100)	156	(100)	237	(100)	508	(100)
Type of antibiotic*												
Penicillin V or pivmecillinam	5	(9.1)	1	(5.9)	0	(0)	0	(0)	0	(0)	6	(1.2)
Amoxicillin	5	(9.1)	0	(0)	1	(2.3)	0	(0)	4	(1.7)	10	(2.0)
Amoxicillin + clavulanic acid	1	(1.8)	0	(0)	3	(7)	11	(7.1)	26	(11)	41	(8.1)
Fosfomycin	12	(21.8)	1	(5.9)	0	(0)	5	(3.2)	119	(50.2)	137	(27)
Nitrofurantoin	4	(7.3)	0	(0)	20	(46.5)	56	(35.9)	8	(3.4)	88	(17.3)
Trimethoprim ± sulfonamide	5	(9.1)	0	(0)	4	(9.3)	3	(1.9)	7	(3)	19	(3.7)
Macrolides or clindamycin	2	(3.6)	0	(0)	0	(0)	4	(2.6)	1	(0.4)	7	(1.4)
Cephalosporins	15	(27.3)	5	(29.4)	12	(27.9)	5	(3.2)	29	(12.2)	66	(13.0)
Quinolones	7	(12.7)	6	(35.3)	6	(14)	64	(41)	33	(13.9)	116	(22.8)
Other antibiotics	0	(0)	4	(23.5)	1	(2.3)	36	(23.1)	13	(5.5)	54	(10.6)

*More than one antibiotic may be prescribed; therefore, columns may summarize to more than 100%

### Appropriateness of antibiotic start-up treatment decisions and the national first choice of antibiotics for UTIs

*Appropriateness of antibiotic start-up treatment decisions:* Table 3 presents the quality indicators for antibiotic treatment decisions for UTIs in residents without indwelling urinary catheters. The decision to start antibiotics for UTIs in residents without catheters was deemed highly appropriate for 31% and highly inappropriate for 49% of the residents. The highly inappropriate antibiotic treatment decisions for UTIs vary across countries: France (38%), Lithuania (28%), Poland (10%), and Spain (68%). Table 4 presents the results of the linear mixed model regression analyzing the association between country and appropriateness of antibiotic start-up treatment decisions. Compared to France, no significant difference was in the appropriateness of the antibiotic start-up treatment decision in Lithuania (0.118, *ρ* = 0.764). However, a significant negative correlation between France and Poland (-0.639, *ρ* = 0.024), while a significant positive correlation was observed between France and Spain (0.864, *ρ* < 0.001). These findings indicate that antibiotic start-up treatment decisions for UTIs were more appropriate in Poland and less appropriate in Spain than in France.

**Table 3:** Appropriateness of antibiotic start-up treatment for UTIs and choice of antibiotic type for UTIs among residents in nursing homes

		France***		Greece*****	Lithuania****		Poland*****		Spain****		Total	
Appropriateness of antibiotic start-up treatment decision		n	%		n	%	n	%	n	%	n	%
Highly appropriate	≥ 2 lower UTI symptoms* OR flank/back pain	16	(38.1)	N/A	6	(33.3)	50	(72.5)	30	(15.1)	102	(31.1)
Likely appropriate	1 lower UTI symptom AND one of systemic symptoms**	1	(2.4)	N/A	1	(5.6)	5	(7.3)	3	(1.5)	10	(3.0)
Likely inappropriate	1 lower UTI symptom* and no systemic symptoms**	9	(21.4)	N/A	6	(33.3)	7	(10.1)	31	(15.6)	53	(16.2)
Highly inappropriate	No UTI symptoms	16	(38.1)	N/A	5	(27.8)	7	(10.1)	135	(67.8)	163	(49.7)
Total		42	(100)	N/A	18	(100)	69	(100)	199	(100)	328	(100)
Appropriateness of the choice of antibiotic type												
First-line antibiotic, n (%)		13	(28.3)	N/A	12	(38.7)	33	(30.8)	118	(55.4)	176	
Total		46	(100)	N/A	31	(100)	107	(100)	213	(100)	397 (100)	

*Lower UTI symptoms: dysuria, urgency, frequency, incontinence (18)

**Systemic symptoms: fever or shaking chills

***National first-line antibiotic for cystitis: Fosfomycin, amoxicillin

****National first-line antibiotics for cystitis: Fosfomycin, nitrofurantoin

*****National first-line antibiotics for cystitis: Fosfomycin, nitrofurantoin, trimethoprim + sulfamethoxazole

**Table 4:** Results from linear mixed model regression assessing differences between countries in the appropriateness of antibiotic start-up treatment for UTI among residents without a urinary catheter, *n* = 328

	Coefficient (95% CI)*	P-value
Intercept	2.615 (2.147;3.082)	
Country		
France	Ref	
Lithuania	0.118 (–0.657; 0.892)	0.764
Poland	–0.639 (–1.192; 0.085)	0.024
Spain	0.864 (0.365; 1.362)	< 0.001

*Adjusted for age and sex

*National first-line antibiotics for UTIs:* In Spain and Lithuania, 55% and 39% of antibiotic treatments for UTIs, respectively, are national first-line antibiotics. Moreover, 30% and 28% of antibiotic treatments for UTIs in Poland and France, respectively, use first-line antibiotics.

## Discussion

### Summary of key results

This study identified differences in antibiotic prescribing patterns for suspected UTIs in nursing homes across five European countries. The proportion of antibiotics prescribed for UTIs ranged from 23 to 59% of all antibiotics used in the target countries. Notably, 49% of these prescriptions were given to residents without UTI-specific symptoms. The prevalence of highly inappropriate antibiotic treatment decisions varied widely, from 10% in Poland to 68% in Spain. In addition, less than half of the antibiotic treatments for UTIs involve a first-line antibiotic. The use of first-line antibiotics varies considerably, from 28% in France to 55% in Spain.

### Findings in relation to other studies

Variation in antibiotic use in nursing homes within and across European countries has previously been demonstrated [11]. In this study, only approximately 2–4% of antibiotics prescribed for UTIs were given to prevent infection, which is lower than the percentage reported in point prevalence studies [11, 20]. This difference is expected because point prevalence studies are more likely to observe preventive treatments because they are administered over a longer course than start-up treatments.

This study focused on the quality of antibiotic start-up treatments for UTIs. We found that in nursing homes in Spain, antibiotics were given more often for a UTI than for other infection types. This finding is in line with a study from 2016 that reported that the use of antibiotics in nursing homes was relatively high in Spain compared with other European countries [11]. We also measured the inappropriateness of antibiotic treatments for UTIs on the basis of the required presence of UTI-specific symptoms. Spain had the highest proportion of inappropriate antibiotic treatment decisions for UTIs compared with the other four countries. Similarly, other studies have examined the use of antibiotics for asymptomatic bacteriuria, for which antibiotics are not appropriate, and have reported the frequent use of antibiotics for asymptomatic bacteriuria in nursing homes [21].

There may be several explanations for the observed differences across countries. In this study, we observed some differences in the resident populations across countries. Prior studies have implied that differences in health and functional status among nursing home residents are not a driving force behind the appropriate prescription of antibiotics for UTIs [11, 21]. Rather, organizational and cultural factors may play a role in the observed variation in antibiotic treatment among residents at nursing homes [11]. For example, studies have focused on the use and interpretation of diagnostic tests [22]. In practice, a wide range of events, such as a change in cognitive status, behavior, a change in color or smell of urine, or even a fall, may prompt the ordering of urine tests [22]. Given the high prevalence of asymptomatic bacteriuria (the presence of bacteria in urine from a resident but not having symptoms of UTI), a high proportion of urine tests will be positive regardless of symptoms [23]. Studies have shown that positive urine test results play an important role in the decision to initiate antibiotics despite the inability of a urine test to discriminate between asymptomatic bacteriuria and symptomatic infection [24].

### Strengths and limitations

To our knowledge, this is the first study to provide detailed information on and compare the appropriateness of antibiotic use for UTIs in nursing homes across European countries. The data are restricted to the clinical description predefined in the APO chart, which has shown high reliability in various European projects and correlates well with actual prescribing [15].

One of the primary limitations of this study is the small number of participating nursing homes across the five countries. The limited sample size significantly constrains the ability to perform robust comparative statistical analyses, as it reduces the statistical power and the ability to detect meaningful differences in antibiotic use among countries. Consequently, our analyses are primarily descriptive in nature. Furthermore, we did not obtain sufficient data from Greece to include them in the analyses.

Another limitation of this study is the selection of nursing homes on the basis of motivation. Given that nursing homes that agree to participate might already be interested in the rational diagnosis and treatment of UTIs, the proportion of inappropriate use of antibiotics for UTIs may be even greater in other nursing homes in these countries. Furthermore, the data came from nursing homes that varied in relation to the medical services available and the dependency level of the residents. However, we argue that the quality indicator in relation to symptom presentation is relevant to all the resident populations included in the analyses, even though the diagnosis of a UTI may be more difficult in residents with cognitive impairment.

The data collection took place from February to April 2022 during the 5th COVID-19 wave. Even though the consumption of antibiotics decreased during the COVID-19 pandemic, the number of antibiotic treatments for UTIs among older adults decreased less, because UTIs are less strongly associated with circulating transmissible infections.

We applied criteria for symptom presentation when assessing the antibiotic treatment decision for UTIs on the basis of a Delphi consensus study [18]. Different quality indicators exist [19, 25, 26], including the widely known (revised) McGeer criteria that have been developed for surveillance rather than for clinical decision-making. To our knowledge, there are no internationally validated or nationally validated quality indicators for assessing the quality of the diagnostic process and treatment decision for UTIs in the nursing home population. We chose the criteria used by van Buul et al. [18] to guide the data collection and assessment of the appropriateness of antibiotic treatment decision for UTIs in residents without urinary catheters. The criteria used by van Buul et al. [18] are similar to the other criteria in the sense that they focus on the presence of genitourinary symptoms for UTI diagnosis. Furthermore, looking only at the population without indwelling urinary catheters allowed us to compare the appropriateness of the antibiotic treatment decision. However, this decision also neglects the potential for improvement among residents with urinary catheters. Indeed, 40% and 46% of antibiotic treatments for UTIs were administered to residents with a urinary catheter in Lithuania and Poland, respectively. This study does not provide an explanation for the observed differences in the appropriateness of choice of antibiotic type for suspected UTIs. Notably, data on urine culture results were not collected, meaning we cannot assess the extent to which prescribers’ antibiotic choices were influenced by these results. Incorporating urine culture data could have provided a stronger basis for evaluating the appropriateness of antibiotic selection.

### Implications for practice

The EU guidelines for the prudent use of antimicrobials in human health state that a multifaceted approach should be established in long-term care, which includes elements such as education of nursing and medical staff, audits of antimicrobial use, feedback to prescribers, and targeting identified areas of antimicrobial overuse and misuse [3]. Our results identify targets for future antimicrobial stewardship interventions in nursing homes, with a focus on specific symptoms indicating UTIs, appropriate use of diagnostic tests, and the choice of antibiotics. The role of nurses is critical within the clinical team because of their regular contact with patients and their role in administering medicines [3]. Moreover, the prescribing doctor is responsible for the decision to use antibiotics and choose the type of antibiotics [3]. Future research should investigate explanations for differences in appropriate antibiotic use for UTIs, such as cultural and organizational factors (e.g., use and interpretation of diagnostic tests and communication between the nursing home staff and the prescriber doctor). Furthermore, our study underlines the need for more knowledge about the correct (combination of) symptoms that indicate UTIs in the older adult population.

## Conclusion

This study highlights differences in antibiotic prescribing practices for suspected urinary tract infections in nursing homes across five European countries. Despite the known guidelines for prudent antibiotic use, our findings indicate a substantial proportion of inappropriate antibiotic treatment.

Antimicrobial stewardship programs in nursing homes must be strengthened to reduce inappropriate antibiotic use. Future research should further explore the underlying reasons for these differences and develop tailored interventions to improve antibiotic prescribing practices in nursing homes.

## Supplementary Information

Below is the link to the electronic supplementary material.

Supplementary file1 (PDF 278 KB)

## Data Availability

The data that support the findings of this study are available upon request from the corresponding author. The data are not publicly available due to privacy or ethical restrictions.

## Abbreviations

APO: Audit project odense
UTI: Urinary tract infection
