# Correction: Appropriateness of antibiotic use in nursing homes for suspected urinary tract infections: comparison across five European countries

**DOI:** 10.1007/s41999-025-01300-1

**Published:** 2025-09-11

**Authors:** Matilde Bøgelund Hansen, Jesper Lykkegaard, Malene Plejdrup Hansen, Carl Llor, Ana Garcia Sangenis, Pia Touboul-Lundgren, Pascale Bruno, Ruta Radzeviciene, Lina Jaruseviciene, Marilena Anastasaki, Christos Lionis, Anna Kowalczyk, Maciek Godycki-Ćwirko, Beatriz Gonzalez Lopez-Valcárcel, Fabiana Raynal Floriano, Athina Chalkidou, Lars Bjerrum, Jette Nygaard Jensen

**Affiliations:** 1 Department of Clinical Microbiology, Copenhagen University Hospital–Herlev and Gentofte, Copenhagen, Denmark; 2 Audit Project Odense, Research Unit of General Practice, University of Southern Denmark, Odense, Denmark; 3 Center for General Practice, Aalborg University, Aalborg, Denmark; 4 Institut Català de La Salut, Via Roma Health Centre, C. Manso, 19, 3rd Floor, 08015 Barcelona, Spain; 5 Fundació Institut Universitari Per a La Recerca a L’Atenció Primària de Salut Jordi Gol, Barcelona, Spain; 6 Department of Public Health, Nice University Hospital, Nice, France; 7 Ltd. Mano Seimos Gydytojas (My Family Doctor), Klapeida, Lithuania; 8 Lithuanian University of Health Sciences, Kaunas, Lithuania; 9 Clinic of Social and Family Medicine, School of Medicine, University of Crete, Rethymn, Greece; 10 Centre for Family and Community Medicine, The Faculty of Health Sciences, The Medical University of Lodz, Lodz, Poland; 11 University of Las Palmas de Gran Canaria, Las Palmas, Spain; 12 Section of General Practice and Research Unit for General Practice, Department of Public Health, University of Copenhagen, Copenhagen, Denmark; 13 Capital Region Committee for Antimicrobial Stewardship and Prevention of Hospital Infections, Hillerød, Denmark

**Correction: European Geriatric Medicine (2025) 16:1453–1464** 10.1007/s41999-025-01185-0

In the original version of this article, the given and family names of the author Bruno Pascale were interchanged.

Correct version of given and family names of the author are:

Pascale Bruno

The original article has been corrected.

