# Supplementary material for: Appropriateness of antibiotic use in nursing homes for suspected urinary tract infections: comparison across five European countries
Source: Eur Geriatr Med. 2025 Mar 27;16(4):1453–64. doi: 10.1007/s41999-025-01185-0 (PMC12378316; doi:10.1007/s41999-025-01185-0)
Supplement: Supplementary file 1 — Supplementary file1 (PDF 278 KB) [file 41999_2025_1185_MOESM1_ESM.pdf]

Participant number:
9-9-99

HAPPY PATIENT-1:
Infections in nursing homes (resident receiving antibiotics)

	Age (years)	Sex		Focus of infection<br>Only 1 X				Non specific symptoms<br>Min 1 X				Urogenital symptoms<br>Min 1 X									Indwelling urinary catheter?<br>Only 1 X		Diagnostic tests<br>Min 1 X			Antibiotics (only systemic)<br>Min 1 X										Type of treatment<br>Only 1 X				Treatment started when resident was at<br>Only 1 X			Treatment duration	Perceived demand for antibiotics<br>Only 1 X		
		F	M	Urinary tract	Respiratory tract	Skin	Other	Fever (temp. ≥ 38 °C)	Shaking chills	Confusion	None of the above	Painful urination	Urgency	Frequency	Urinary incontinence	Flank/back pain	Obvious blood in urine	Foul smelling urine	Cloudy urine	None of the above	Yes	No	Urinary dipstick performed	Urine culture sent to lab	None of the above	Penicillin V or pivmecillinam	Amoxicillin	Amoxicillin + clavulanic acid	Fosfomycin	Nitrofurantoin	Trimethoprim +/- sulfonamide	Macrolides or clindamycin	Cephalosporins	Quinolones	Other antibiotics	Startup treatment	Prophylaxis (preventive)	Extension of current treatment	Unknown	Nursing home	Hospital	Unknown	Treatment duration – days<br>999=unknown<br>888=continuous	From resident or relative	From staff	No perceived demand
				1	2	3	4	5	6	7	8	9	10	11	12	13	14	15	16	17	18	19	20	21	22	23	24	25	26	27	28	29	30	31	32	33	34	35	36	37	38	39	40	41	42	43
1																																														
2																																														
3																																														
4																																														
5																																														
6																																														
7																																														
8																																														

NH-UK

®Copyright: Audit Projekt Odense, J.B. Winsløws Vej 9A, 1. 5000 Odense C
